# Use of Paracervical Blocks for Patients Who Undergo Intrauterine Device Insertion

**DOI:** 10.1001/jamanetworkopen.2026.8406

**Published:** 2026-04-21

**Authors:** Jacquelyn M. Roger, Jean Costello, Halle Young, Thaybeth I. Malave-Mendez, Brenda Y. Miao, Claire D. Brindis, Karen Meckstroth, Ryan D. Hernandez, Sara Whetstone, Marissa Raymond-Flesch, John A. Capra, M. Maria Glymour, Dara Torgerson

**Affiliations:** 1 Biological and Medical Informatics Graduate Program, University of California, San Francisco; 2 Division of Research, Kaiser Permanente Northern California, Pleasanton; 3 Department of Environmental Health, Boston University, Boston, Massachusetts; 4 Medical Anthropology Graduate Program, University of California, San Francisco; 5 Pharmaceutical Sciences and Pharmacogenomics Graduate Program, University of California, San Francisco; 6 Bakar Computational Health Sciences Institute, University of California, San Francisco; 7 Philip R. Lee Institute for Health Policy Studies, University of California, San Francisco; 8 Department of Obstetrics, Gynecology, and Reproductive Sciences, University of California, San Francisco; 9 Division of Adolescent and Young Adult Medicine, University of California, San Francisco; 10 Department of Bioengineering and Therapeutic Sciences, University of California, San Francisco; 11 Department of Epidemiology and Biostatistics, University of California, San Francisco; 12 Department of Epidemiology, Boston University, Boston, Massachusetts

## Abstract

**Importance:**

Paracervical blocks can reduce pain during intrauterine device (IUD) insertions, but the extent and context in which they are used in practice are unclear.

**Objective:**

To evaluate paracervical block use during IUD insertions in an academic health system.

**Design, Setting, and Participants:**

This retrospective cross-sectional time-series study was performed at the University of California, San Francisco (UCSF), academic health care system using electronic health record data. Participants included individuals who underwent IUD insertions between July 2, 2012, and August 16, 2024. Data were analyzed from October 2023 to April 2025.

**Exposure:**

Clinic-, clinician-, and patient-level variables.

**Main Outcomes and Measures:**

Paracervical block receipt was determined from clinical notes. Associations of paracervical block receipt with variables at the clinic, clinician, and patient levels were estimated with multilevel models. The role of clinic specialty (pediatrics or adolescent medicine vs all other specialties) in accounting for the association of age less than 18 years with paracervical block receipt was evaluated using causal mediation analysis.

**Results:**

Among 13 702 IUD insertions (median patient age, 33.16 [IQR, 27.62-37.73] years), 11 713 (85.5%) took place within obstetrics and gynecology (OB-GYN) specialty clinics and almost half (6049 [44.1%]) occurred between a patient-clinician dyad who had never seen each other before. Overall, 5311 insertions (38.8%) included a paracervical block. Paracervical blocks were less frequently used in non–OB-GYN specialties (odds ratio [OR], 0.01; 95% CI, 0.001-0.07) and appointments scheduled for less than 30 minutes (OR, 0.77; 95% CI, 0.66-0.90) and more frequently used when the patient-clinician dyad had a prior visit together (OR, 1.27; 95% CI, 1.09-1.48). A lower proportion of patients aged less than 18 years (84 of 347 [24.2%]) compared with adults (5227 of 135 55 [38.6%]) received a paracervical block, an association mediated by the former population more often receiving care in pediatrics and adolescent medicine clinics (total effect risk difference [RD], −0.11 [95% CI, −0.19 to −0.03]; indirect effect RD, −0.22 [95% CI, −0.26 to −0.18]). Latine patients (OR, 0.77; 95% CI, 0.64-0.94 [reference group, non-Hispanic White patients]) and patients with a preferred language besides English (OR, 0.63; 95% CI, 0.41-0.96) had lower odds of receiving a paracervical block during IUD insertion.

**Conclusions and Relevance:**

In this cross-sectional time series study of IUD insertion procedures in a large academic health system, Latine ethnicity, non-English language preference, no prior patient-clinician visit together, and shorter appointment length were associated with lower odds of receiving paracervical blocks. Potential conduits for increasing paracervical block access include training opportunities, equitable clinic policies, and sufficient appointment duration.

## Key Points

**Question:**

How often and in which contexts are paracervical blocks included in intrauterine device (IUD) insertion procedures?

**Findings:**

In this cross-sectional study of 13 702 IUD insertions at a large academic health system, a paracervical block was used in 5311 insertions (39%), with wide variation across specialties and clinics. Rates of paracervical block use were lower among clinicians in pediatrics and adolescent medicine specialties.

**Meaning:**

These findings suggest that supporting clinicians’ access to training and modifying clinic infrastructures (eg, supplies and guidelines) may improve patients’ access to pain control in this common gynecologic procedure.

## Teaser

This cross-sectional study investigates the use of paracervical blocks during intrauterine device insertion procedures among patients within an academic health system and assesses the associations of paracervical block receipt with variables at the clinic, clinician, and patient levels.

## Introduction

An intrauterine device (IUD) is an essential medical technology used for contraception, emergency contraception, gender-affirming care, cancer treatment, and management of chronic gynecologic conditions.^1,2,3,4,5,6^ While there are many benefits of IUDs, a significant downside is that their insertion procedures cause moderate or even severe pain in a substantial portion of patients.^7,8,9,10^ In a survey of 620 patients who underwent IUD insertions,^11^ nearly half (41.6%) reported experiencing an unacceptable level of pain during the procedure. This pain unnecessarily burdens patients, creates barriers to one of the most effective forms of contraception, and sows medical distrust. Fears around IUD insertion pain are especially prevalent among adolescents and young adults.^12^ In a survey of 46 adolescents,^13^ 41% shared that their dislike of their insertion procedure would maybe or probably prevent them from getting an IUD again in the future. In another survey of 1982 female undergraduate students,^14^ 30% reported that worries about insertion pain were a barrier to using an IUD. A study of social media discourse^15^ found that 28% of most-liked videos about IUDs mentioned distrust of health care professionals. These factors may deter patients from getting an IUD in instances when it might otherwise offer them optimal care for their needs.

Many strategies have been explored to reduce IUD insertion pain, and some of the most promising results have come from studies of paracervical blocks. Paracervical blocks using lidocaine are an evidence-based strategy to reduce pain with IUD insertion.^16^ Several randomized clinical trials with different populations have shown that paracervical blocks are beneficial for reducing pain throughout the procedure, particularly among nulliparous individuals.^17,18,19,20,21,22,23^ In one trial of nulliparous adolescents and young adults undergoing IUD insertion procedures,^20^ those receiving a paracervical block reported a median pain level of 30.0 of 100 at IUD placement compared with 71.5 of 100 in the control arm. In another trial of nulliparous patients,^22^ the median pain reported at IUD insertion was 33 of 100 with a paracervical block vs 54 of 100 in the control arm.

Current clinical guidelines from the Centers for Disease Control and Prevention suggest a benefit of paracervical blocks; however, the guidelines do not provide a strong recommendation for their routine use, either overall or for specific patients (eg, nulliparous individuals).^24^ The American College of Obstetricians and Gynecologists (ACOG) recently released guidance that more strongly states the value of paracervical blocks for IUD insertions and recommends that clinicians discuss and offer pain control options for the procedure.^25^ Findings from prior studies suggest that the overall use of paracervical blocks for IUD insertion procedures in the US has been low: Two nationwide studies^26,27^ reported near-zero rates of paracervical block use. However, smaller studies from the US and Canada^28,29,30^ found that a number of their clinicians performing IUD insertions offered paracervical blocks and called for better training and elimination of logistical barriers to pain control.

In this study, our objective was to examine paracervical block use during IUD insertion procedures within the University of California, San Francisco (UCSF), an academic health system with more than 100 clinical locations around the San Francisco Bay Area.^31^ This study occurs at a salient time, given the updated recommendations by ACOG and recent clinician-led efforts to increase training and access to paracervical blocks for IUD insertions in 2 different clinics at UCSF.

## Methods

This cross-sectional study was approved by the Institutional Review Board at UCSF, which waived the need for informed consent because the research was no more than minimal risk and could not practicably be carried out otherwise. We followed the Strengthening the Reporting of Observational Studies in Epidemiology (STROBE) reporting guideline.

### Study Population

**Figure 1.**
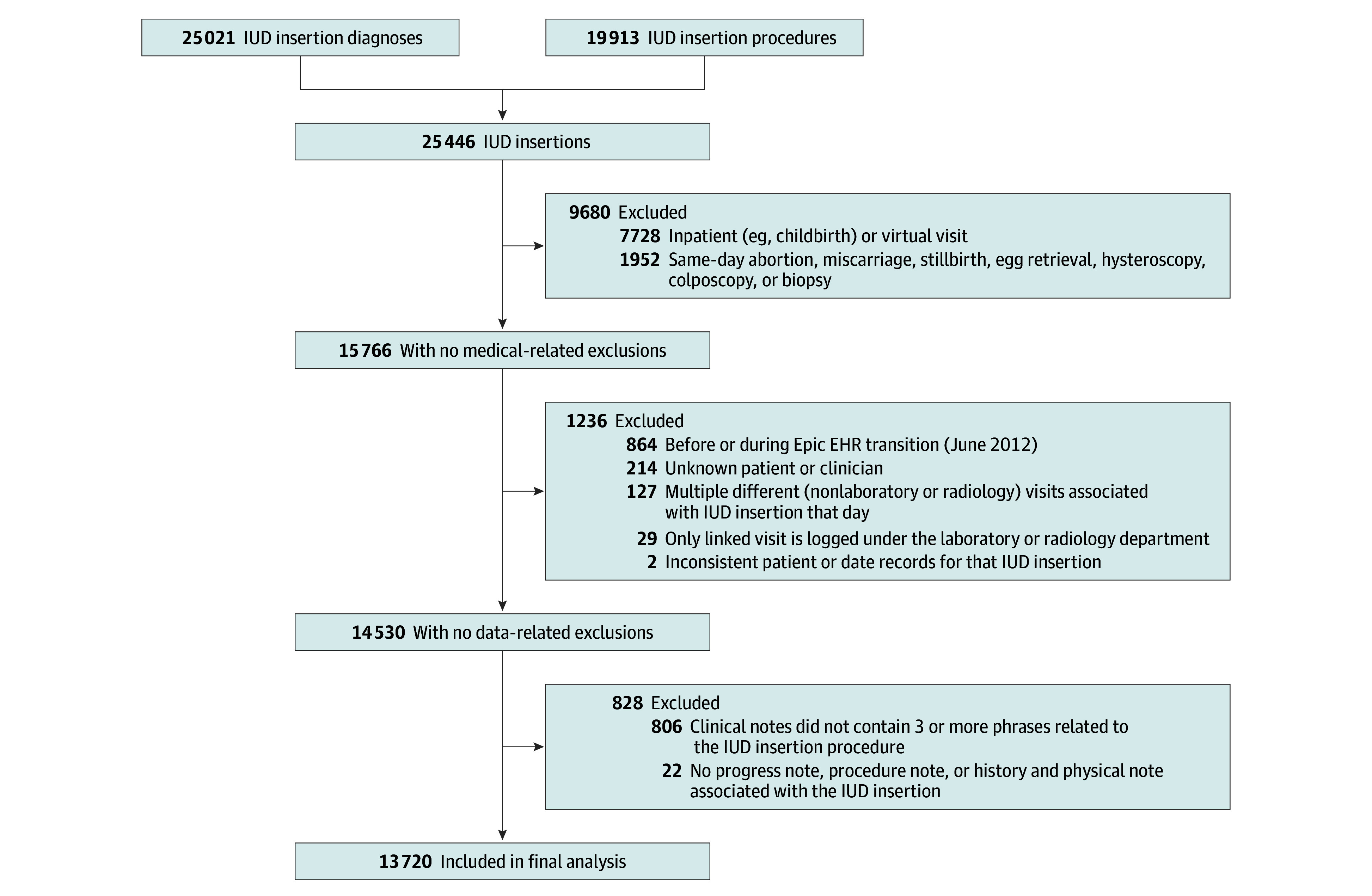
Flowchart of Intrauterine Device (IUD) Insertion Querying and Filtering EHR indicates electronic health record.

UCSF’s electronic health records (EHRs) contain deidentified data dating back to 1982, with the bulk of the records after June 2012, when UCSF transitioned to its current EHR system (Apex [Advanced Patient-Centered Excellence] based on Epic Systems Corporation product). There were more than 6 million patients and more than 161 million encounters as of August 2024. We queried the Limited Data Set of the Clinical Data Warehouse for diagnoses and procedures corresponding to IUD insertions; all included IUD insertions were then subject to medical- and data-related exclusion criteria (Figure 1 and eMethods and eAppendix in Supplement 1). Race and ethnicity were derived from self-identified race and ethnicity in the EHR database (eMethods in Supplement 1) and were included in the analysis in order to assess whether paracervical block receipt varied by the race or ethnicity of the patient. Racial and ethnic categories included Asian, Black, Latine, White, multiracial or multiethnic, unknown or declined, and other (including American Indian or Alaska Native, Native Hawaiian or Other Pacific Islander, North African, Southwest Asian, and other).

### Determining Paracervical Block Receipt From Clinical Notes

Paracervical block receipt is more reliably recorded in unstructured clinical notes compared with structured medications or procedures data. We therefore determined paracervical block receipt for each IUD insertion by analyzing its associated clinical notes using a rule-based decision tree whose design was informed by clinicians who regularly perform IUD insertions at UCSF (eMethods, eFigures 1 and 2, and eAppendix in Supplement 1). A subset of 200 IUD insertions was then randomly subsampled for manual review to validate our decision tree (eMethods in Supplement 1).

### Statistical Analysis

Descriptive statistics for each variable were calculated using χ^2^ tests or Fisher exact tests (when any expected cell count was <5). To account for between-clinic and between-clinician heterogeneity in paracervical block use, paracervical block receipt was modeled using a multilevel logistic regression model (model 1). The explanatory variables were all clinic-, clinician-, and patient-level variables as well as year (eMethods in Supplement 1). Race and ethnicity were included as a patient-level variable. The response variable was paracervical block receipt. Random intercepts were fitted for clinics and clinicians and clinics.

Odds ratios (ORs) for all variables, adjusted for covariates, were estimated from the multilevel model. The multilevel model was implemented with the glmer() function in the lme4 R package^32^ with the following parameters: bobyqa optimizer, maximum of 100 000 function evaluations in model fitting, and a random-effects model structure of clinicians nested within clinics. We calculated variance partition coefficients (VPCs) using the latent response formulation to estimate the proportion of unexplained variance in paracervical block use attributed to each level.^33^

To test whether relative differences in paracervical block receipt between age groups changed over time, we fit another multilevel model that included all of the variables in model 1 plus a linear year trend variable and an interaction between age group and the linear year trend (model 2). To test whether relative differences in paracervical block receipt between racial and ethnic groups changed over time, we fit a similarly structured model for race and ethnicity (model 3).

Differences in paracervical block use patterns across clinics or clinicians may give rise to patient-level differences in paracervical block receipt. Compared with adults, a higher proportion of patients younger than 18 years seek IUD care within pediatrics and adolescent medicine specialties. To investigate the extent that age-specific differences in paracervical block receipt may be mediated by medical specialty differences, we performed a causal mediation analysis. Our hypothesized causal framework for this analysis is represented in a directed acyclic graph in eFigure 3 in Supplement 1. The exposure variable was age less than 18 years (binary true or false), the mediator variable was receiving care in a clinic specializing in pediatrics or adolescent medicine (binary true or false), and the outcome variable was paracervical block receipt (binary true or false). Using causal assumptions and the analytic approach outlined by Imai et al^34^ to generate simulated counterfactual contrasts, the total effect estimate of age less than 18 years on paracervical block receipt was decomposed into the direct effect of age less than 18 years and the indirect effect mediated through pediatrics and adolescent medicine (eMethods in Supplement 1).

Data were analyzed from October 2023 to April 2025. Two-sided *P* < .05 was considered statistically significant. All analyses were performed using R, version 4.0.2 (R Project for Statistical Computing).

## Results

### Baseline Characteristics 

**Figure 2.**
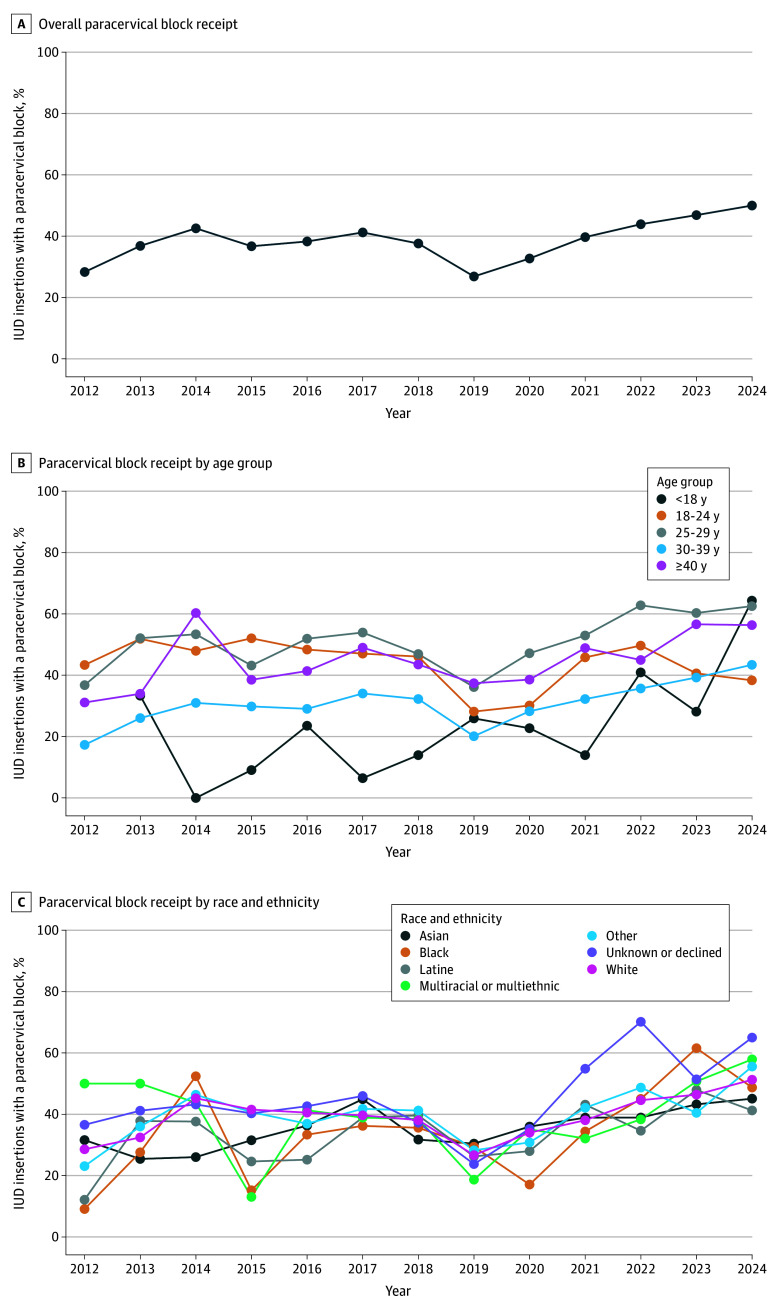
Line Graphs of Paracervical Block Receipt Over Time IUD indicates intrauterine device.

Our analytic sample included 13 702 IUD insertions among 11 503 patients (median patient age, 33.16 [IQR, 27.62-37.73] years) from July 2, 2012, through August 16, 2024 (Figure 1). Baseline characteristics are provided in Table 1. Most IUD insertions (11 713 [85.5%]) took place within obstetrics and gynecology (OB-GYN) specialty clinics. Almost half of IUD insertions (6049 [44.1%]) occurred between a patient-clinician dyad who had never seen each other before. In terms of age distribution, 347 patients (2.5%) were younger than 18 years, 1783 (13.0%) were aged 18 to 24 years, 2572 (18.8%) were aged 25 to 29 years, 6714 (49.0%) were aged 30 to 39 years, and 2286 (16.7%) were 40 years or older. In terms of race and ethnicity, 1907 patients (13.9%) were Asian, 596 (4.3%) were Black, 1670 (12.2%) were Latine, 5163 (37.7%) were White, 556 (4.1%) were multiracial or multiethnic, 1647 (12.0%) were of other race or ethnicity, and 2163 (15.8%) were of unknown race or ethnicity or declined to provide this information. Overall, 5311 procedures (38.8%) included a paracervical block (Figure 2A). Paracervical block classification was concordant with results of manual review for 196 of 200 IUD insertions (98.0%) in the benchmarked subsample (eResults in Supplement 1). The use of paracervical blocks during IUD insertions increased from 2012 to 2024, with fluctuations year to year (Figure 2). Of the 62 clinics represented in our analytic sample, 18 had at least 100 IUD insertions. Among those, within-clinic paracervical block use ranged from 0 to 89% (median, 32% [IQR, 1%-50%]). Of the 346 clinicians, 100 had performed at least 20 IUD insertions. Among those, within-clinician use of paracervical block ranged from 0 to 98% (median, 4% [IQR, 0-46%]).

**Table 1.** Patient Characteristics

Characteristic	Patient group, No. (%)^a^			*P* value^b^
	Without paracervical block (n = 8391)	With paracervical block (n = 5311)	Overall (N = 13 702)	
Medical specialty				
OB-GYN	6572 (56.1)	5141 (43.9)	11 713 (100)	<.001
Other	80 (64.5)	44 (35.5)	124 (100)	
Pediatrics or adolescent medicine	557 (100)	0	557 (100)	
Primary care or family medicine	1182 (90.4)	126 (9.6)	1308 (100)	
Appointment near end of shift	1765 (66.1)	904 (33.9)	2669 (100)	<.001
Appointment scheduled for <30 min	2085 (76.0)	659 (24.0)	2744 (100)	<.001
Appointment started ≥10 min late				
Yes	2505 (63.9)	1414 (36.1)	3919 (100)	<.001
Unknown	50 (69.4)	22 (30.6)	72 (100)	
Type of clinician				
Attending physician	4250 (61.5)	2660 (38.5)	6910 (100)	<.001
Midwife	971 (99.0)	10 (1.0)	981 (100)	
Nurse practitioner	2682 (52.7)	2404 (47.3)	5086 (100)	
Resident physician	470 (66.7)	235 (33.3)	705 (100)	
Other or unknown or declined	18 (90.0)	2 (10.0)	20 (100)	
Gender of clinician				
Men	368 (85.8)	61 (14.2)	429 (100)	<.001
Women	7745 (60.4)	5074 (39.6)	12 819 (100)	
Nonbinary, unknown, or declined	278 (61.2)	176 (38.6)	454 (100)	
Clinician’s previous IUD insertions				
<50	4127 (75.0)	1378 (25.0)	5505 (100)	<.001
50-200	3024 (63.7)	1725 (36.3)	4749 (100)	
>200	1240 (36.0)	2208 (64.0)	3448 (100)	
No. of times clinician has seen patient				
0	3527 (58.3)	2522 (41.7)	6049 (100)	<.001
1	1491 (55.2)	1212 (44.8)	2703 (100)	
>1	3373 (68.1)	1577 (31.9)	4950 (100)	
Patient age group, y				
<18	263 (75.8)	84 (24.2)	347 (100)	<.001
18-24	1003 (56.3)	780 (43.7)	1783 (100)	
25-29	1267 (49.3)	1305 (50.7)	2572 (100)	
30-39	4629 (68.9)	2085 (31.1)	6714 (100)	
≥40	1229 (53.8)	1057 (46.2)	2286 (100)	
Patient race and ethnicity				
Asian	1205 (63.2)	702 (36.8)	1907 (100)	<.001
Black	375 (62.9)	221 (37.1)	596 (100)	
Latine	1088 (65.1)	582 (34.9)	1670 (100)	
White	3139 (60.8)	2024 (39.2)	5163 (100)	
Multiracial or multiethnic	347 (62.4)	209 (37.6)	556 (100)	
Other^c^	998 (60.6)	649 (39.4)	1647 (100)	
Unknown or declined	1239 (57.3)	924 (42.7)	2163 (100)	
Patient preferred language				
English	8156 (60.9)	5242 (39.1)	13 398 (100)	<.001
Other than English	233 (78.2)	65 (21.8)	298 (100)	
Unknown or declined	2 (33.3)	4 (66.7)	6 (100)	
Patient had a previous birth	4508 (81.0)	1060 (19.0)	5568 (100)	<.001
Patient within 6 mo post partum	3868 (86.4)	608 (13.6)	4476 (100)	<.001

Abbreviations: IUD, intrauterine device; OB-GYN, obstetrics and gynecology; UCSF, University of California, San Francisco.

^a^ Percentages are calculated by row.

^b^ Calculated using χ^2^ tests or Fisher exact tests (when any expected cell count was <5).

^c^ Includes American Indian or Alaska Native, Native Hawaiian or Other Pacific Islander, North African, Southwest Asian, and other race or ethnicity or categories with low patient counts in the sample.

### Associations With Paracervical Block Receipt

Descriptive statistics are provided in Table 1 and adjusted associations from model 1 are provided in Table 2. Most variation in the probability of a paracervical block being used during an IUD insertion (after accounting for measured covariates) was between clinics (VPC for clinic = 0.55), compared with between clinicians (VPC for clinician = 0.24) or unexplained (VPC for IUD insertion = 0.21). Paracervical block use was higher within OB-GYN specialty clinics (5141 of 11 713 [43.9%]) compared with primary care and family medicine clinics (126 of 1308 [9.6%]) or pediatrics and adolescent medicine clinics (0 of 557). IUD insertions within a non–OB-GYN specialty clinic had much lower odds of receiving a paracervical block (OR, 0.01; CI, 0.001-0.07). Odds of paracervical blocks were lower in appointments scheduled for less than 30 minutes (OR, 0.77; 95% CI, 0.66-0.90) and in appointments near the end of a shift (11 am to 12 pm or 4-5 pm; OR, 0.81; 95% CI, 0.70-0.95). IUD insertions in which the patient-clinician dyad had a prior visit together vs no prior visit was associated with higher odds of a paracervical block (OR, 1.27; 95% CI, 1.09-1.48); this association remained with multiple prior visits, but the odds did not increase (OR, 1.18; 95% CI, 1.02-1.35). Compared with IUD insertions with clinicians who performed fewer than 50 prior IUD insertions (1378 of 5505 [25.0%] of which included a paracervical block), the odds of a paracervical block was greater with clinicians who performed 50 to 200 prior IUD insertions (OR, 2.00; 95% CI, 1.64-2.45) and greatest among those with more than 200 prior IUD insertions (OR, 2.84; 95% CI, 2.06-3.90). Among 3448 IUD insertions performed by a clinician with more than 200 prior IUD insertions, 2208 (64.0%) included a paracervical block. Paracervical block use was lower among nurse practitioners compared with attending physicians (OR, 0.38; 95% CI, 0.19-0.76).

**Table 2.** Results of Multilevel Logistic Regression, Model 1

Variable	OR (95% CI)	*P* value
Medical specialty not OB-GYN	0.01 (0.00-0.07)	<.001
Appointment near end of shift	0.81 (0.70-0.95)	.01
Appointment scheduled for <30 min	0.77 (0.66-0.90)	.001
Appointment started ≥10 min late		
Yes	0.97 (0.86-1.10)	.68
Unknown	0.65 (0.33-1.31)	.23
Type of clinician		
Midwife	0.04 (0.01-0.16)	<.001
Nurse practitioner	0.38 (0.19-0.76)	.01
Other or unknown or declined	11.02 (0.31-392.15)	.19
Resident physician	1.78 (0.86-3.65)	.12
Attending physician	1 [Reference]	NA
Gender of clinician		
Men	0.63 (0.25-1.59)	.32
Women	1 [Reference]	NA
Nonbinary, unknown, or declined	0.96 (0.23-3.97)	.96
Clinician’s previous IUD insertions		
<50	1 [Reference]	NA
50-200	2.00 (1.64-2.45)	<.001
>200	2.84 (2.06-3.90)	<.001
No. of times clinician has seen patient		
0	1 [Reference]	NA
1	1.27 (1.09-1.48)	.003
>1	1.18 (1.02-1.35)	.02
Patient age group, y		
<18	2.14 (1.15-3.97)	.02
18-24	2.06 (1.66-2.56)	<.001
25-29	1.86 (1.55-2.22)	<.001
30-39	1.13 (0.97-1.32)	.11
≥40	1 [Reference]	NA
Patient race and ethnicity		
Asian	0.89 (0.74-1.07)	.21
Black	0.87 (0.65-1.15)	.33
Latine	0.77 (0.64-0.94)	.01
White	1 [Reference]	NA
Multiracial or multiethnic	0.82 (0.61-1.10)	.19
Other^a^	0.98 (0.81-1.18)	.82
Unknown or declined	1.09 (0.92-1.30)	.30
Patient preferred language		
English	1 [Reference]	NA
Other than English	0.63 (0.41-0.96)	.03
Unknown or declined	0.71 (0.08-5.94)	.75
Patient had a previous birth	0.27 (0.22-0.34)	<.001
Patient within 6 mo post partum	0.30 (0.24-0.38)	<.001

Abbreviations: IUD, intrauterine device; NA, not applicable; OB-GYN, obstetrics and gynecology; OR, odds ratio.

^a^ Includes American Indian or Alaska Native, Native Hawaiian or Other Pacific Islander, North African, Southwest Asian, and other race or ethnicity or categories with low patient counts in the sample.

Comparisons by age indicated that a lower proportion of patients younger than 18 years received a paracervical block compared with other age groups (<18 years, 84 of 347 [24.2%]; 18-24 years, 780 of 1783 [43.7%]; 25-29 years, 1305 of 2572 [50.7%]; 30-39 years, 2085 of 6714 [31.1%]; ≥40 years, 1057 of 2286 [46.2%]). However, age less than 18 years was positively associated with paracervical block receipt (OR, 2.14; 95% CI, 1.15-3.97 [reference group, ≥40 years]) in models adjusted for medical specialty. Across racial and ethnic groups, paracervical blocks were included in IUD insertion procedures for 702 of 1907 Asian patients (36.8%), 221 of 596 Black patients (37.1%), 582 of 1670 Latine patients (34.9%), 2024 of 5163 White patients (39.2%), 209 of 556 multiracial or multiethnic patients (37.6%), and 649 of 1647 patients (39.4%) who were of other race or ethnicity. Latine patients had lower odds of receiving a paracervical block compared with White patients (OR, 0.77; 95% CI, 0.64-0.94). Patients who indicated a preferred language other than English had lower odds of receiving a paracervical block during IUD insertion (OR, 0.63; 95% CI, 0.41-0.96).

Results from models 2 and 3 are provided in eTables 1 and 2 in Supplement 1, respectively. At the start of our time series in 2012, odds of paracervical block receipt were higher for the groups aged 18 to 24 years (OR, 6.27; 95% CI, 3.96-9.95) and 25 to 29 years (OR, 3.20; 95% CI, 2.24-4.59) compared with those 40 years or older. Increases during the study period were smaller for the groups aged 18 to 24 years (interaction OR, 0.83; 95% CI, 0.78-0.89) and 25 to 29 years (interaction OR, 0.91; 95% CI, 0.86-0.96) compared with those 40 years or older. Considering temporal trends in racial and ethnic differences in paracervical block receipt, odds of paracervical block in 2012 were lower among Asian patients (OR, 0.49; 95% CI, 0.32-0.74), Black patients (OR, 0.37; 95% CI, 0.20-0.66), and Latine patients (OR, 0.63; 95% CI, 0.42-0.95) compared with White patients. Increases over the years were larger for Asian patients (interaction OR, 1.09; 95% CI, 1.03-1.16) and Black patients (interaction OR, 1.15; 95% CI, 1.06-1.25) but not for Latine patients (interaction OR, 1.03; 95% CI, 0.98-1.09) compared with White patients.

### Causal Mediation Analysis of Patients Younger Than 18 Years and Medical Specialty

Of the 62 clinics represented in our analytic sample, 8 specialized in pediatrics or adolescent medicine. Most IUD insertions for patients younger than 18 years (228 of 347 [65.7%]) took place in pediatrics or adolescent medicine clinics, compared with 329 of 13 355 (2.5%) among adults. The interaction between age less than 18 years and pediatrics and adolescent medicine specialties in the paracervical block receipt model was not significant, so it was not included in the mediation analysis. The estimated total risk difference (RD) for the association of age less than 18 years with paracervical block receipt was 11 percentage points (RD, −0.11; 95% CI, −0.19 to −0.03). This total association was decomposed into a large indirect effect mediated by receiving care in a pediatrics or adolescent medicine clinic (RD, −0.22; 95% CI, −0.26 to −0.18) offset by a positive direct effect (RD, 0.11; 95% CI, 0.02-0.20) that was not mediated by the clinic where care was received.

## Discussion

In this large academic health care system, less than half of IUD insertions from mid-2012 to mid-2024 included a paracervical block. In some clinics, paracervical blocks were never or rarely used, yet in other clinics, paracervical blocks were used for some or even most IUD insertions. Paracervical block use was highest within OB-GYN clinics and lowest in primary care and family medicine clinics; paracervical block was not reported to have occurred in pediatrics and adolescent medicine clinics.

Differences in medical training almost certainly contribute to the observed specialty-related variation. For example, OB-GYN training is more likely to include paracervical blocks for various procedures. Pediatricians can provide a trusted and accessible option for IUD insertion for adolescents, but have few other reasons to learn paracervical blocks and are not necessarily taught them as part of any IUD training. In a recent mixed-methods study of clinicians’ viewpoints on pain management for IUD insertions,^28^ some clinicians expressed frustration about a lack of available paracervical block training. A potential strategy for improving patients’ access to paracervical blocks is to increase clinicians’ training opportunities, particularly among non–OB-GYN specialties. For example, one UCSF adolescent medicine clinic organized a training session where a gynecologist was brought in to teach about safe administration of paracervical blocks; subsequently, clinicians in that clinic began offering paracervical blocks to their patients undergoing IUD insertion procedures. Another UCSF-affiliated OB-GYN clinic trained all advanced practice clinicians in performing paracervical blocks with two 1-hour in-person sessions in 2023; the clinic then experienced an increase in use within months.

Several clinical factors may affect paracervical block receipt for patients undergoing IUD insertion procedures, including appointment timing and duration, the patient-clinician relationship, and the clinician’s level of experience with the procedure. Paracervical blocks were less frequently used in appointments scheduled for less than 30 minutes and in appointments near the end of a shift. Time-of-day effects have been reported in other areas of medicine,^35,36,37^ and when present, may signal the presence of structural time constraints that are not conducive to patient-centered clinical decision-making. Clinicians report that logistical concerns, such as time, are indeed barriers to providing pain relief during IUD insertions.^29^ Paracervical blocks were more often used during IUD insertions with clinicians who had an established relationship with a patient (ie, clinician had previously seen the patient before), and with clinicians more experienced with performing IUD insertions. This is consistent with the well-established link between clinical volume and health care quality.^38^

We observed some notable disparities in paracervical block receipt by age and racial and ethnic identity. Patients younger than 18 years received paracervical blocks less than all other age groups, which is likely due to receiving IUD care in pediatrics and adolescent medicine specialty clinics that had no recorded use of paracervical blocks. Latine patients had lower odds of receiving a paracervical block compared with White patients, even after adjusting for other potential correlates. Additional studies are needed to better understand this disparity. Asian and Black patients did not have lower odds of a paracervical block than White patients overall; however, receipt at the beginning of our time series (2012) was higher for White patients. Subsequently, use of paracervical block increased over time most quickly for Asian and Black patients. Additional studies are required to rule out the possibility that our observed temporal differences were not simply due to chance year-to-year fluctuations in paracervical block use, although UCSF has implemented multiple antiracism training sessions for clinicians during this period, which may explain this change. Racial and ethnic disparities in access to pain medication have been documented throughout many areas of medicine, including among children and adolescents.^39,40,41,42,43,44^ Qualitative dissemination and implementation studies among clinicians regarding the barriers to implementing paracervical blocks for IUD insertions may help identify conduits for positive change. One potential conduit for equity in access to paracervical blocks is establishing clear policies involving all stakeholders to set a common standard of care accessible to all patients. For example, a UCSF-affiliated OB-GYN clinic created a practice guideline recommending paracervical blocks for all IUD insertions.

Experiences of untreated or dismissed pain can cause trauma and disillusionment with the health care system.^45,46,47,48,49^ There are long-standing concerns about pain in IUD insertion procedures, and addressing this pain is a priority for patients.^11,15^ The existing evidence on paracervical blocks for IUD insertions is promising, and we anticipate that broader discussions on this topic will continue to grow and evolve, particularly in light of the recently updated ACOG guidance advocating for universally discussing pain control with patients for office-based gynecologic procedures.^25^ Reflecting these clinical practice trends, we documented that thousands of IUD insertions at UCSF included paracervical blocks, with the highest prevalence in OB-GYN clinics and among clinicians who have the most extensive experience performing IUD insertion procedures.

### Limitations

This study has some limitations. UCSF is an academic health system with access to specialty care (including many clinicians with specialized training in complex family planning specifically), and it is not necessarily representative of other health systems. There is some evidence that a 3-mL intracervical block may also reduce pain in the procedure; this alternative may be implemented more easily in some practice settings and should also be explored.^50^ We cannot delineate between instances when a patient was not offered a paracervical block vs when a paracervical block was offered but declined. Some births outside UCSF are not recorded in UCSF data, so whether a patient was parous or post partum was potentially underestimated in our analyses, particularly in the earlier years of the dataset. Similarly, data on obstetric history, including parity and history of vaginal vs cesarean deliveries, are not routinely collected in the EHR data, so they could not be incorporated in our analyses. Interpretation of our mediation analysis rests on 5 assumptions (eMethods in Supplement 1) that we believe are likely to be largely met. Two possible threats to these assumptions are the potential undermeasured parity and postpartum confounding and the possibility that parity and postpartum status could be influenced by age less than 18 years.

## Conclusions

In this cross-sectional study of 13 702 IUD insertions at UCSF, paracervical block use was 38.8%, with wide variation across specialties and clinics. Paracervical block receipt was lower among patients younger than 18 years, Latine patients, and patients whose preferred language was not English. This is an evolving situation, both at UCSF and in the broader medical community. For clinics and clinicians who wish to increase paracervical block access, potential conduits include increasing access to paracervical block training for clinicians, setting clinic policies or guidelines (and subsequent infrastructures) that support equity in paracervical block access for patients, and ensuring that there is sufficient time for patient-centered care in IUD appointments.

## Notes

Supplement 1. **eMethods.** Inclusion and Exclusion Criteria and Analyses **eResults.** Benchmarking IUD Insertions and Paracervical Block Receipt and Robustness Check
eReferences
**eTable 1.** Model 2 Results **eTable 2.** Model 3 Results **eFigure 1.** Clinical Notes Before Processing **eFigure 2.** Paracervical Block Decision Tree **eFigure 3.** Directed Acyclic Graph of Hypothesized Causal Framework for Mediation Analysis **eAppendix.** Included and Excluded Diagnoses and Procedures, IUD Insertion Common Phrases, and Paracervical Block Medications and Procedures

Supplement 2.
Data Sharing Statement
