# Supplementary material for: Use of Paracervical Blocks for Patients Who Undergo Intrauterine Device Insertion
Source: JAMA Netw Open. 2026 Apr 21;9(4):e268406. doi: 10.1001/jamanetworkopen.2026.8406 (PMC13100842; doi:10.1001/jamanetworkopen.2026.8406)
Supplement: Supplement 1. — eMethods. Inclusion and Exclusion Criteria and Analyses eResults. Benchmarking IUD Insertions and Paracervical Block Receipt and Robustness Check eReferences eTable 1. Model 2 Results eTable 2. Model 3 Results eFigure 1. Clinical Notes Before Processing eFigure 2. Paracervical Block Decision Tree eFigure 3. Directed Acyclic Graph of Hypothesized Causal Framework for Mediation Analysis eAppendix. Included and Excluded Diagnoses and Procedures, IUD Insertion Common Phrases, and Paracervical Block Medications and Procedures [file jamanetwopen-e268406-s001.pdf]

Supplementary Online Content

Roger JM, Costello J, Young H, et al. Use of paracervical block for patients who undergo intrauterine device insertion. *JAMA Netw Open.* 2026;9(4):e268406. doi:10.1001/jamanetworkopen.2026.8406

eMethods. Inclusion and Exclusion Criteria and Analyses
eResults. Benchmarking IUD Insertions and Paracervical Block Receipt and Robustness Check
eReferences
eTable 1. Model 2 Results
eTable 2. Model 3 Results
eFigure 1. Clinical Notes Before Processing
eFigure 2. Paracervical Block Decision Tree
eFigure 3. Directed Acyclic Graph of Hypothesized Causal Framework for Mediation Analysis
eAppendix. Included and Excluded Diagnoses and Procedures, IUD Insertion Common Phrases, and Paracervical Block Medications and Procedures

This supplementary material has been provided by the authors to give readers additional information about their work.

© 2026 Roger JM et al. *JAMA Network Open*.

eMethods. Inclusion and Exclusion Criteria and Analyses

IUD insertion inclusion and exclusion criteria

To identify IUD insertions in UCSF EHR, we queried for diagnoses and procedures containing “intrauterine device”, “intrauterine contraceptive device”, or “IUD” and “insert” or “place”. The full lists of included diagnoses and procedures (following manual review to filter out irrelevant items) are in the eAppendix section below. All queries for diagnoses and procedures were based on their names (not codes) to prioritize query specificity because the codes in this EHR database are many-to-many mapped. Following the initial inclusion criteria, IUD insertions were excluded if they took place in an inpatient setting (eg, around childbirth) or occurred on the same day as another medical procedure (eg, abortion) that could necessitate equal or greater pain management (eAppendix). IUD insertions were also excluded if the patient or clinician were unknown or the procedure was logged under multiple visits. Lastly, all procedure notes, progress notes, and history & physical notes that were linked to these IUD insertions were queried, and then IUD insertions were filtered to ensure there was at least one clinical note describing the procedure. IUD insertions with at least one clinical note containing at least three common procedure-related phrases (eg, “cleansed cervix”) were retained (eAppendix). All other clinical notes that were not describing the procedure were excluded from downstream clinical notes analyses.

Paracervical block decision tree analysis

Before applying the decision tree, clinical notes were pre-processed to standardize text formatting, address UCSF-specific idiosyncrasies, detect and correct important misspellings of key terms related to paracervical blocks, and concatenate notes into one combined note per IUD insertion. Misspelling detection was implemented using fuzzy string matching with the find() function in the stringdist R package[1]. Additional details on pre-processing are in eFigure 1. After pre-processing, all IUD insertions’ clinical notes were processed using the decision tree, where each decision-point in the tree was implemented using regular expressions with the str_detect() function in the stringr R package[2]. A high-level summary of the decision tree is: any cervical block that was not explicitly intracervical was estimated to be a paracervical block, and any lidocaine that was injected paracervically (eg, “at 4 and 8 o’clock” of the cervix) or of higher volume (ie, 6+ cc) was also estimated to be a paracervical block. If none of those scenarios were present in the clinical notes, it was estimated that a paracervical block was not included (details in eFigure 2).

Benchmarking IUD insertions and paracervical blocks

A set of 200 IUD insertions were randomly subsampled for manual confirmation of whether an IUD insertion was truly occurring in that visit, whether the procedure was successfully completed, and whether the patient received a paracervical block. Annotations were based on human review of the raw (not pre-processed) clinical notes to evaluate the possibility that the pre-processing could have accidentally introduced errors into the paracervical block determinations. Procedure completion was assessed to evaluate the possibility that the procedure did not sufficiently progress for there to be an opportunity for a paracervical block. Paracervical block determinations were made using the same logic as outlined in the decision tree (ie, our objective was to assess how accurate the computational implementation of the decision tree was, not to assess potential variability in paracervical block definitions). Additionally, IUD patients’ medications and procedures data were queried to also compare paracervical block results from structured data. The full lists of medications and procedures corresponding to paracervical blocks are in the eAppendix.

Explanatory variables in Model One

Clinic-level variables included medical specialty, whether the appointment was near the end of the standard shift hours (either between 11am-12pm for end of morning shift or between 4pm-5pm for end of afternoon shift), whether the appointment was scheduled for less than 30 minutes, and whether the appointment started 10 or more minutes late (estimated by subtracting the time the patient was roomed from the scheduled appointment start time). Clinician-level variables included type, gender, number of previous IUD insertions (in this dataset), and whether the clinician had previously seen the patient. Patient-level variables included age group, race/ethnicity, preferred language (dichotomized as English or not English), previous birth (at UCSF), and within 6 months postpartum from a birth that occurred at UCSF. For age group, we chose the oldest group (40+ years old) as a referent. For race/ethnicity, we chose White as a referent to assess for any health disparities in paracervical block receipt for

© 2026 Roger JM et al. *JAMA Network Open*.

historically underserved patient populations. The race/ethnicity variable in UCSF's EHR database is derived from a combination of self-identified race, self-identified ethnic group, and self-identified ethnicity, using a framework developed by the Health Equity Division, Data Equity Taskforce, and Health Equity Council at UCSF. Their approach first consolidates ethnic group and ethnicity information and then incorporates race to form nine derived race/ethnicity categories including a multi-race/ethnicity category and an other race ethnicity-category. Of note: for patients who identified as Black and answered "yes" to "Hispanic or Latino", their derived race/ethnicity was Latinx; for patients who identified as White and answered "yes" to "Hispanic or Latino", their derived race/ethnicity was Latinx. For our analyses, we used their derived race/ethnicity variable with two modifications: "Latinx" was updated to "Latine" and racial/ethnic categories with low patient counts (Native American or Alaska Native, Native Hawaiian or other Pacific Islander, and Southwest Asian and North African) were combined with the category of other race/ethnicity. (Latine is a gender-neutral term for Latina or Latino[31].) Indicator variables for year of appointment were also included.

Structural forms for model 1, model 2, and model 3

Model 1:
paracervical_block ~ obgyn_clinic + appt_end_of_shift + appt_less30 + appt_10mins_late +
clinician_type + clinician_gender + clinician_previous_iuds + clinician_previously_seen_patient +
patient_age_group + patient_race_ethnicity_bin + patient_preferred_language +
previous_birth + within_6months_postpartum +
year +
(1 | clinic_name / clinician_id)

Model 2:
paracervical_block ~ obgyn_clinic + appt_end_of_shift + appt_less30 + appt_10mins_late +
clinician_type + clinician_gender + clinician_previous_iuds + clinician_previously_seen_patient +
patient_age_group + patient_race_ethnicity_bin + patient_preferred_language +
previous_birth + within_6months_postpartum +
year +
decades_since_july2012 + patient_age_group:decades_since_july2012 +
(1 | clinic_name / clinician_id),

Model 3:
paracervical_block ~ obgyn_clinic + appt_end_of_shift + appt_less30 + appt_10mins_late +
clinician_type + clinician_gender + clinician_previous_iuds + clinician_previously_seen_patient +
patient_age_group + patient_race_ethnicity_bin + patient_preferred_language +
previous_birth + within_6months_postpartum +
year +
decades_since_july2012 + patient_race_ethnicity_bin:decades_since_july2012 +
(1 | clinic_name / clinician_id)

Note: Effect estimates for the linear year trend were transformed from the "decades since july 2012" (beginning of time series) variable, which was utilized to facilitate model convergence.

Mediation analysis

Our causal estimands were estimated under the following five assumptions: no unmeasured exposure-outcome confounding, no unmeasured mediator-outcome confounding, no unmeasured exposure-mediator confounding, no mediator-outcome confounding affected by exposure, and no mediator measurement error. First, a model predicting receiving care at a pediatrics or adolescent medicine clinic was fit as a function of minor age and confounders. Second, a model for the outcome of receiving a paracervical block was fit using minor age, pediatrics and adolescent medicine, and confounders as independent variables. In both models, confounders included race/ethnicity, preferred language, previous birth, postpartum status, and year of care. An interaction between minor age and receiving care in a pediatrics or adolescent medicine clinic was tested for significance and, if applicable, included in the paracervical block receipt model. Then, true and false observations for minor age were simulated for the full IUD insertion dataset, and counterfactual contrasts were generated by predicting out mediator and outcome values using

© 2026 Roger JM et al. *JAMA Network Open*.

the fitted models. The total, direct, and indirect effects were estimated by calculating mean differences in probability of paracervical block receipt between relevant counterfactual contrasts. Point estimates and 95% confidence intervals for effects were estimated nonparametrically via bootstrapping (1000 iterations).

eResults. Benchmarking IUD Insertions and Paracervical Block Receipt and Robustness Check

Benchmarking IUD insertions and paracervical block receipt

A random subsample of 200 IUD insertions were manually annotated based on their clinical notes to serve as a benchmark for our computational analyses. In this subsample, all 200 were confirmed as IUD insertion procedures, and 194 (97%) were successfully completed. For all 6 uncompleted IUD insertions, the procedures were stopped midway through, confirming that they had sufficiently progressed for there to be an opportunity for a paracervical block. When compared with manual annotations, paracervical block results were concordant for 196 (98%) out of 200 IUD insertions in the subsample. Upon manual re-review of the 4 discordances, 3 were human annotation errors and 1 was a computational (decision tree analysis) error. Paracervical blocks were not reliably logged in the structured data: paracervical block results between structured (medications and procedures) data and manual annotations were 76% concordant. Using the manual annotations as a gold-standard, less than half (43%) of paracervical blocks in the subsample were recorded in the structured data.

Robustness check for multilevel model

The allFit() function was used to check the robustness of the multilevel model results to optimizer selection. A maximum of 100,000 function iterations was selected. Overall, results were quite robust to all possible optimizers. Across the fixed effect variables, coefficients were identical to the tenth decimal place for nearly all variables except unknown/declined clinician type, year 2022, and year 2024, of which all were within one-tenth.

eReferences

1. Loo MPJ van der. The stringdist Package for Approximate String Matching. *R J*. 2014;6(1):111-122.

2. Wickham H. stringr: Simple, Consistent Wrappers for Common String Operations. Published online November 9, 2009:1.5.1. doi:10.32614/CRAN.package.stringr

© 2026 Roger JM et al. *JAMA Network Open*.

eTable 1. Model 2 Results

Variable	Odds Ratio	Confidence Interval	p-value
**Medical specialty**			
OB-GYN	(ref)		
Not OB-GYN	0.01	0.00, 0.07	0.00
**Appointment near end of shift**			
Yes	0.83	0.71, 0.96	0.02
**Appointment scheduled for <30 mins**			
Yes	0.76	0.65, 0.89	0.00
**Appointment started 10+ minutes late**			
Yes	0.97	0.85, 1.10	0.60
Unknown	0.62	0.31, 1.26	0.19
**Type of clinician**			
Midwife	0.04	0.01, 0.15	0.00
Nurse Practitioner	0.38	0.19, 0.78	0.01
Other or Unknown/Declined	10.47	0.28, 386.90	0.20
Resident Physician	1.80	0.87, 3.71	0.11
Attending Physician	(ref)		
**Gender of clinician**			
Women	(ref)		
Men	0.62	0.24, 1.58	0.32
Nonbinary or Unknown/Declined	1.01	0.24, 4.17	0.99
**Clinician's previous IUD insertions**			
Less than 50 previous IUD insertions	(ref)		
Between 50-200 previous IUD insertions	1.97	1.61, 2.41	0.00
More than 200 previous IUD insertions	2.69	1.95, 3.71	0.00
**Whether clinician has seen patient before**			
Never seen patient before	(ref)		
Seen patient once before	1.26	1.08, 1.48	0.00
Seen patient more than once before	1.20	1.04, 1.38	0.01
**Patient's age group**			
<18	7.94	0.90, 70.51	0.06
18-24	6.27	3.96, 9.95	0.00
25-29	3.20	2.24, 4.59	0.00
30-39	0.99	0.72, 1.35	0.92
40+	(ref)		
**Patient's race/ethnicity**			
Asian	0.89	0.75, 1.07	0.22

© 2026 Roger JM et al. *JAMA Network Open*.

Black	0.85	0.64, 1.14	0.28
Latine	0.77	0.63, 0.93	0.01
Multi-Race/Ethnicity	0.83	0.62, 1.11	0.21
Other	0.98	0.81, 1.18	0.82
Unknown/Declined	1.06	0.89, 1.26	0.49
White	(ref)		
**Patient's preferred language**			
English	(ref)		
Not English	0.63	0.42, 0.97	0.04
Unknown/Declined	0.62	0.07, 5.33	0.66
**Patient has a previous birth (at UCSF)**			
Yes	0.26	0.21, 0.31	0.00
**Patient is within 6 months postpartum (at UCSF)**			
Yes	0.31	0.24, 0.39	0.00
**Linear year trend**	0.98	0.80, 1.19	0.81
**Interaction between patient's age group and linear year trend**			
<18 x linear year trend	0.84	0.65, 1.09	0.19
18-24 x linear year trend	0.83	0.78, 0.89	0.00
25-29 x linear year trend	0.91	0.86, 0.96	0.00
30-39 x linear year trend	1.03	0.98, 1.07	0.24
40+ x linear year trend	(ref)		

Other race/ethnicity includes patients identified as other race/ethnicity, as well as racial/ethnic categories with low patient counts in the sample, including Native American or Alaska Native, Native Hawaiian or other Pacific Islander, and Southwest Asian and North African.

eTable 2. Model 3 Results

Variable	Odds Ratio	Confidence Interval	p-value
**Medical specialty**			
OB-GYN	(ref)		
Not OB-GYN	0.01	0.00, 0.07	0.00
**Appointment near end of shift**			
Yes	0.82	0.70, 0.96	0.01
**Appointment scheduled for <30 mins**			
Yes	0.76	0.65, 0.89	0.00
**Appointment started 10+ minutes late**			
Yes	0.98	0.86, 1.10	0.69
Unknown	0.67	0.33, 1.34	0.26
**Type of clinician**			
Midwife	0.04	0.01, 0.16	0.00
Nurse Practitioner	0.38	0.19, 0.77	0.01
Other or Unknown/Declined	11.46	0.32, 412.55	0.18
Resident Physician	1.81	0.88, 3.73	0.11
Attending Physician	(ref)		
**Gender of clinician**			
Women	(ref)		
Men	0.62	0.24, 1.57	0.31
Nonbinary or Unknown/Declined	0.96	0.23, 3.95	0.95
**Clinician's previous IUD insertions**			
Less than 50 previous IUD insertions	(ref)		
Between 50-200 previous IUD insertions	2.01	1.64, 2.46	0.00
More than 200 previous IUD insertions	2.84	2.06, 3.92	0.00
**Whether clinician has seen patient before**			
Never seen patient before	(ref)		
Seen patient once before	1.27	1.08, 1.48	0.00
Seen patient more than once before	1.19	1.03, 1.36	0.02
**Patient's age group**			
<18	2.10	1.13, 3.91	0.02
18-24	2.06	1.66, 2.55	0.00
25-29	1.85	1.54, 2.21	0.00
30-39	1.13	0.97, 1.32	0.13
40+	(ref)		
**Patient's race/ethnicity**			
Asian	0.49	0.32, 0.74	0.00

© 2026 Roger JM et al. *JAMA Network Open*.

Black	0.37	0.20, 0.66	0.00
Latine	0.63	0.42, 0.95	0.03
Multi-Race/Ethnicity	0.85	0.43, 1.70	0.65
Other	1.32	0.87, 1.99	0.19
Unknown/Declined	1.19	0.87, 1.62	0.27
White	(ref)		
**Patient's preferred language**			
English	(ref)		
Not English	0.64	0.42, 0.97	0.04
Unknown/Declined	0.69	0.08, 5.74	0.73
**Patient has a previous birth (at UCSF)**			
Yes	0.27	0.22, 0.33	0.00
**Patient is within 6 months postpartum (at UCSF)**			
Yes	0.29	0.23, 0.37	0.00
**Linear year trend**	0.94	0.76, 1.15	0.52
**Interaction between patient's race/ethnicity and linear year trend**			
Asian x linear year trend	1.09	1.03, 1.16	0.00
Black x linear year trend	1.15	1.06, 1.25	0.00
Latine x linear year trend	1.03	0.98, 1.09	0.27
Multi-Race/Ethnicity x linear year trend	1.00	0.91, 1.09	0.91
Other x linear year trend	0.95	0.89, 1.01	0.09
Unknown/Declined x linear year trend	0.97	0.92, 1.02	0.26
White x linear year trend	(ref)		

Other race/ethnicity includes patients identified as other race/ethnicity, as well as racial/ethnic categories with low patient counts in the sample, including Native American or Alaska Native, Native Hawaiian or other Pacific Islander, and Southwest Asian and North African.

© 2026 Roger JM et al. *JAMA Network Open*.

eFigure 1. Clinical Notes Before Processing

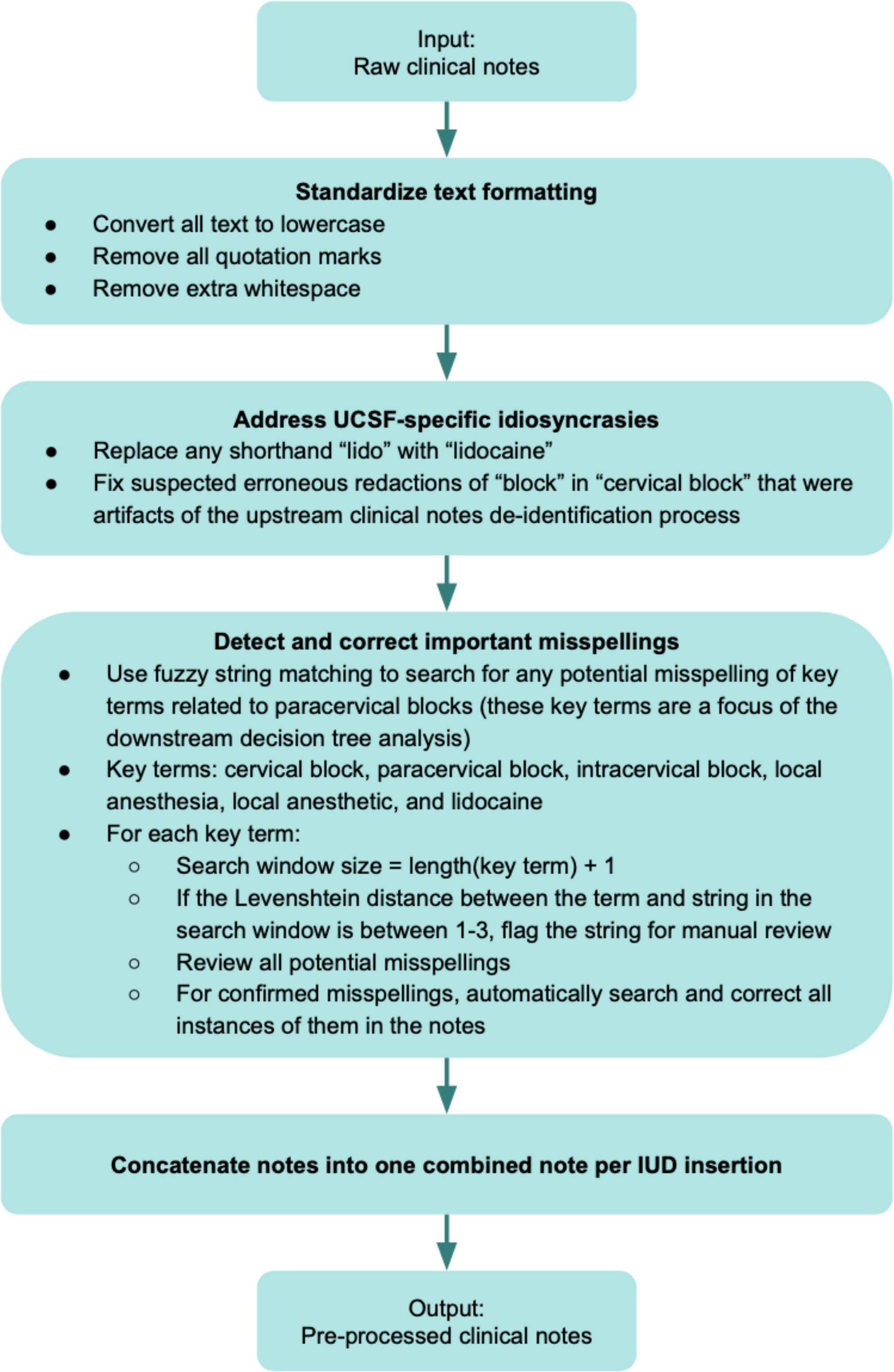

© 2026 Roger JM et al. *JAMA Network Open*.

eFigure 2. Paracervical Block Decision Tree

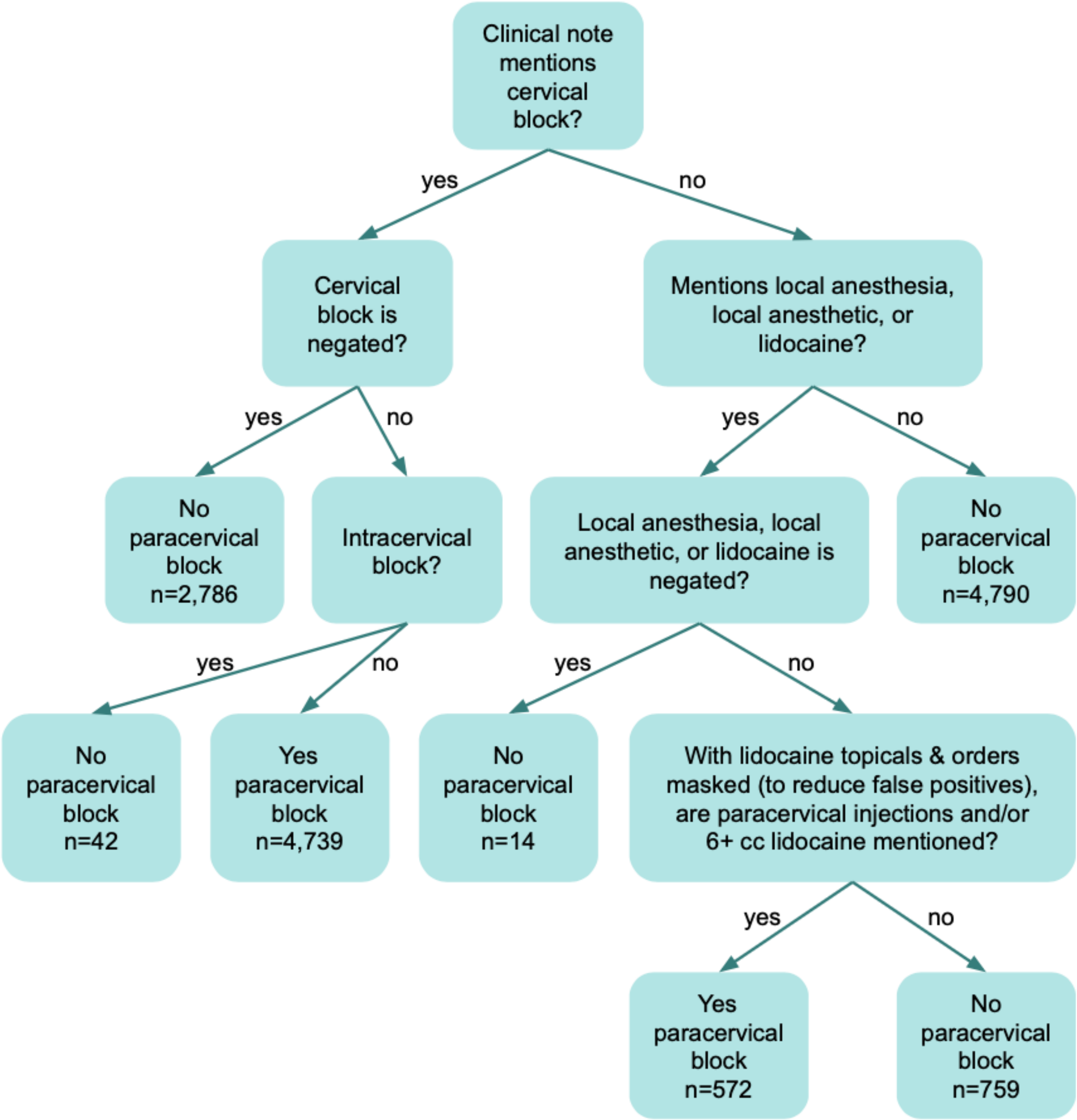

© 2026 Roger JM et al. *JAMA Network Open*.

eFigure 3. Directed Acyclic Graph of Hypothesized Causal Framework for Mediation Analysis

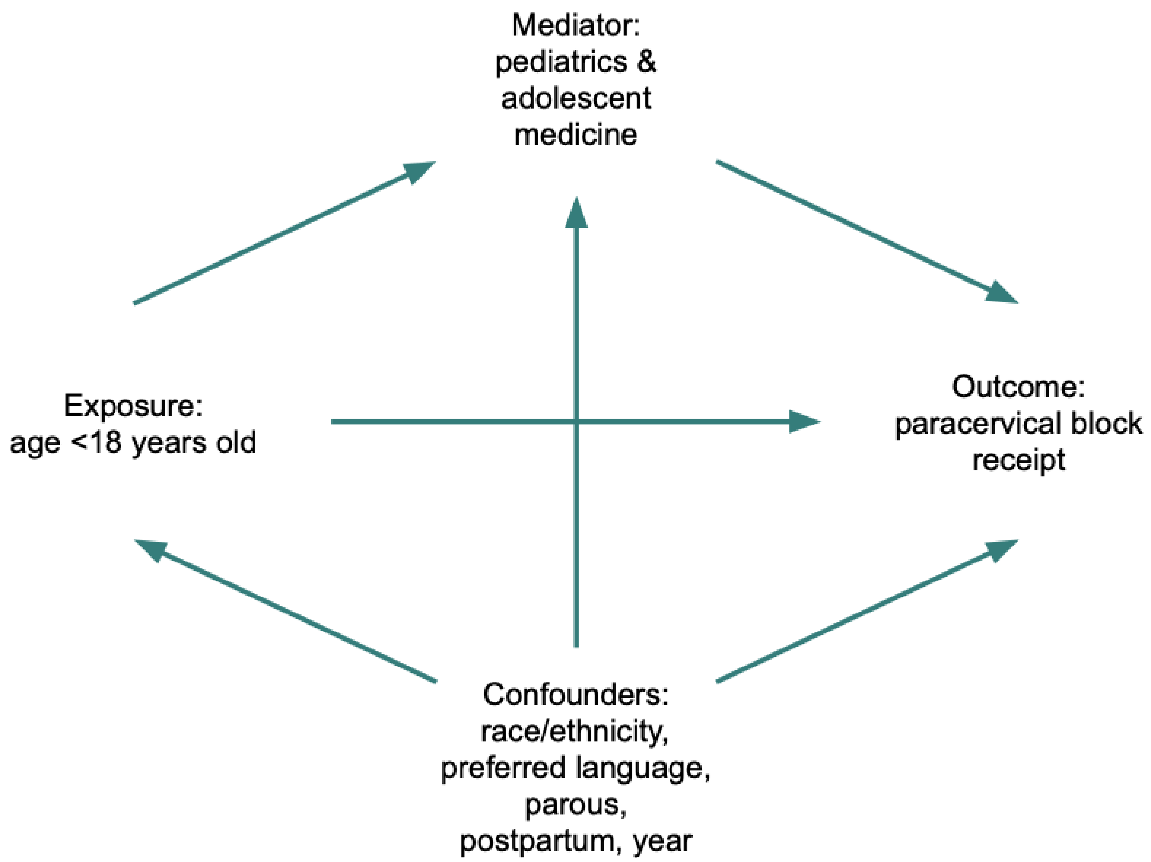

© 2026 Roger JM et al. *JAMA Network Open*.

eAppendix. Included and Excluded Diagnoses and Procedures, IUD Insertion Common Phrases, and Paracervical Block Medications and Procedures

Included diagnoses: diagnoses corresponding to an IUD insertion procedure
Encounter for initial insertion of intrauterine contraceptive device
Encounter for insertion of copper intrauterine contraceptive device (IUD)
Encounter for insertion of copper IUD
Encounter for insertion of intrauterine contraceptive device
Encounter for insertion of intrauterine contraceptive device (IUD)
Encounter for insertion of intrauterine contraceptive device (IUD) for non-contraception indication
Encounter for insertion of mirena IUD
Encounter for insertion of ParaGard IUD
Encounter for insertion of progestin-releasing intrauterine contraceptive device
Encounter for insertion of progestin-releasing intrauterine contraceptive device (IUD)
Encounter for intrauterine device placement
Encounter for IUD insertion
Encounter for IUD removal and reinsertion
Encounter for removal and reinsertion of intrauterine contraceptive device
Encounter for removal and reinsertion of intrauterine contraceptive device (IUD)
Encounter for removal and reinsertion of IUD
Encounter for replacement of intrauterine contraceptive device
Family planning, IUD (intrauterine device) insertion
Insertion of intrauterine contraceptive device
Insertion of intrauterine device
Insertion of intrauterine device (IUD)
Insertion of IUD
Insertion, intrauterine contraceptive device
Visit for insertion of intrauterine device
Visit for IUD coil insertion

Included procedures: Procedures corresponding to an IUD insertion procedure
GYN IUD INSERTION
INSERTION OF IUD
INTRAUTERINE DEVICE INSERTION
PR INSERT INTRAUTERINE DEVICE
PR IUC INSERTION W/MIRENA IUD
PR IUC INSERTION W/PARAGARD IUD

Excluded diagnoses: Diagnoses that, if present on the same day, excluded the IUD insertion from the analytic sample
(induced) termination of pregnancy with other complications
(induced) termination of pregnancy with unspecified complications
Abortion
Abortion complicated by delayed or excessive hemorrhage
Abortion in first trimester
Abortion in second trimester
Abortion on demand
Abortion, elective or therapeutic
Abortion, incomplete
Abortion, legal
Abortion, therapeutic incomplete
Abortion, therapeutic, first trimester
Abortion, therapeutic, second trimester
Complete abortion

© 2026 Roger JM et al. *JAMA Network Open*.

Complete abortion without complication
Complete legal abortion
Complete legal abortion with no complication
Complete legally induced abortion
Complete legally induced abortion complicated by delayed or excessive hemorrhage
Complete legally induced abortion complicated by embolism
Complete legally induced abortion complicated by genital tract and pelvic infection
Complete legally induced abortion without complication
Complete legally induced abortion without mention of complication(635.92)
Complete miscarriage
Complete or unspecified spontaneous abortion without complication
Complete spontaneous abortion
Complete spontaneous abortion with other specified complications
Complete spontaneous abortion without mention of complication
Damage to pelvic organs following (induced) termination of pregnancy
Delayed or excessive hemorrhage following (induced) termination of pregnancy
Delayed or excessive hemorrhage following abortion or ectopic and molar pregnancies
Delayed or excessive hemorrhage following complete or unspecified spontaneous abortion
Delayed or excessive hemorrhage following failed attempted termination of pregnancy
Delayed or excessive hemorrhage following incomplete spontaneous abortion
Elective abortion
Embolism following (induced) termination of pregnancy
Embolism following complete or unspecified spontaneous abortion
Encounter for abortion with abortifacient medication
Encounter for biopsy
Encounter for elective termination of pregnancy
Encounter for fertility preservation procedure
Encounter for fertility preservation procedure prior to cancer therapy
Encounter for medical abortion
Encounter for termination of pregnancy with abortifacient medication
Endometrial cancer determined by uterine biopsy (CMS code)
Failed attempted abortion without mention of complication
Failed attempted medical abortion
Failed attempted termination of pregnancy with other complications
Failed attempted termination of pregnancy without complication
Failed medical abortion, without complication
Fetal death from pregnancy termination
Fetal demise
Fetal demise before 20 weeks with retention of dead fetus
Fetal demise before 22 weeks with retention of dead fetus
Fetal demise due to miscarriage
First trimester abortion
Follow-up visit after therapeutic abortion
Genital tract and pelvic infection following incomplete spontaneous abortion
High grade squamous intraepithelial lesion (HGSIL), grade 2 CIN, on biopsy of cervix
Hyperplasia of endometrium determined by biopsy
Incomplete abortion
Incomplete legal abortion with delayed or excessive hemorrhage
Incomplete legal abortion with no complication
Incomplete legally induced abortion complicated by delayed or excessive hemorrhage
Incomplete legally induced abortion complicated by genital tract and pelvic infection
Incomplete legally induced abortion with other specified complications
Incomplete legally induced abortion without mention of complication
Incomplete spontaneous abortion
Incomplete spontaneous abortion with complication
Incomplete spontaneous abortion with other complications

© 2026 Roger JM et al. *JAMA Network Open*.

Incomplete spontaneous abortion without complication
Incomplete spontaneous abortion without mention of complication
Induced abortion
Legal abortion
Legal abortion with air embolus
Legal abortion with tear of bladder
Legal abortion without complication
Legal termination of pregnancy
Legally induced abortion
Legally induced abortion complicated by genital tract and pelvic infection
Legally induced abortion without mention of complication
Low grade squamous intraepithelial lesion (LGSIL) on biopsy of cervix
Medical abortion
Medical abortion, incomplete, without complication
Miscarriage
Missed abortion
Missed abortion with fetal demise before 20 completed weeks of gestation
Normal hysteroscopy
Retained products of conception following abortion
S/P D&C (status post dilation and curettage)
S/P dilation and curettage
S/P skin biopsy
SAB (spontaneous abortion)
Second trimester abortion
Single stillbirth
Spontaneous abortion, complete, without mention of complication
Spontaneous miscarriage
Status post colposcopy
Status post D&C
Status post dilation and curettage
Status post elective abortion
Status post hysteroscopy
Status post induced abortion
Status post therapeutic abortion
Termination of pregnancy
Termination of pregnancy (fetus)
Therapeutic abortion
Therapeutic abortion in first trimester
Therapeutic abortion in second trimester
Therapeutic abortion with metabolic disorder
Unspecified abortion, without mention of complication, complete
Unspecified abortion, without mention of complication, incomplete
Unspecified abortion, without mention of complication, unspecified
Unspecified legally induced abortion complicated by delayed or excessive hemorrhage
Unspecified legally induced abortion complicated by genital tract and pelvic infection
Unspecified legally induced abortion with other specified complications
Unspecified legally induced abortion with unspecified complication
Unspecified legally induced abortion without mention of complication
Unspecified spontaneous abortion without mention of complication
Unwanted pregnancy with plans for termination

Excluded procedures: Procedures that, if present on the same day, excluded the IUD insertion from the analytic sample

Abortion of Products of Conception, Vacuum, Via Opening
Abortion of Products of Conception, Via Opening
BREAST BIOPSY WITH NEEDLE WIRE LOCALIZATION

© 2026 Roger JM et al. *JAMA Network Open*.

CERVICAL BIOPSY NEC
CERVICAL DILATION AND EVACUATION OF PRODUCT OF CONCEPTION; >12 WEEKS GESTATION
CERVICAL DILATION AND EVACUATION OF PRODUCT OF CONCEPTION; 1-12 WEEKS GESTATION
CHG BIOPSY, OOCYTE POLAR BODY
CHG TISSUE CULTURE, SKIN/BIOPSY
CLOSED UTERINE BIOPSY
COLPOSCOPY
COMBINED HYSTEROSCOPY DIAGNOSTIC / D&C
CONE BIOPSY COLD KNIFE
CYSTOSCOPY WITH HYDRODISTENSION AND BLADDER BIOPSY
D&C DIAGNOSTIC / THERAPEUTIC
DIAGNOSTIC HYSTEROSCOPY
DIAGNOSTIC HYSTEROSCOPY WITH THERMAL ABLATION
DILATION AND CURETTAGE
DILATION AND CURETTAGE (D&C) CERVICAL BIOPSY
DILATION AND CURETTAGE OF UTERUS
DILATION AND CURETTAGE; CERVICAL CONIZATION
EGD WITH CLOSED BIOPSY
ENDO ADULT COLONOSCOPY WITH BIOPSY
ENDO ADULT EGD WITH BIOPSY
ENDO ADULT ENDOMETRIAL BIOPSY
ENDO ADULT FLEX SIG WITH BIOPSY
ENDO PEDS EGD WITH BIOPSY
ENDOMETRIAL THERMAL ABLATION, POSSIBLE DIAGNOSTIC HYSTEROSCOPY
ENDOMETRIAL BIOPSY
ENDOMETRIUM BIOPSY
EXAMINATION UNDER ANESTHESIA, COLPOSCOPY
HC PUNCH BIOPSY SKIN EA SEP/ADDITIONAL LESION
HYSTEROSCOPY
HYSTEROSCOPY OPERATIVE
HYSTEROSCOPY WITH LYSIS OF INTRAUTERINE ADHESIONS/RESECTION OF INTRAUTERINE SEPTUM.
HYSTEROSCOPY, DILATION AND CURETTAGE
INACTIVE OPERATIVE LAPAROSCOPY AND HYSTEROSCOPY
INDUCED ABORTION
LAPAROSCOPIC EXPLORATION OF ABDOMEN FOR DIAGNOSIS, POSSIBLE BIOPSY
LYMPHATIC STRUCT BIOPSY
MG SPECIMEN CORE BIOPSY, LEFT
MUSCLE BIOPSY ARM
OPERATIVE HYSTEROSCOPY, POSSIBLE RESECTION OF FIBROID
PR ANESTH,HYSTEROSCOPY/VAG BX
PR BIOPSY CERVIX, 1 OR MORE, OR EXCISION OF LESION
PR BIOPSY OF SKIN LESION
PR BIOPSY OF URETHRA
PR BIOPSY OF UTERUS LINING
PR BIOPSY OF VAGINA,SIMPLE
PR BIOPSY VULVA/PERINEUM,ONE LESN
PR COLONOSCOPY W/BIOPSY SINGLE/MULTIPLE
PR COLPOSCOPY,CERVIX W/ADJ VAG,W/LOOP BX
PR COLPOSCOPY,CERVIX W/ADJ VAG,W/LOOP CONIZ
PR COLPOSCOPY,CERVIX W/ADJ VAGINA
PR COLPOSCOPY,CERVIX W/ADJ VAGINA, CURETTAG
PR COLPOSCOPY,CERVIX W/ADJ VAGINA,W/BX
PR D&C AFTER DELIVERY
PR D&C OF CERVIX STUMP
PR DILATION/CURETTAGE,DIAGNOSTIC

© 2026 Roger JM et al. *JAMA Network Open*.

PR EGD TRANSORAL BIOPSY SINGLE/MULTIPLE
PR ENDOMET BIOPSY DONE W/COLPOSCOPY
PR FOLLICLE PUNC,RETRIEVAL OF OOCYTE
PR HYSTEROSCOPY,DX,SEP PROC
PR HYSTEROSCOPY,LYSIS ADHESIONS
PR HYSTEROSCOPY,RESECT SEPTUM
PR HYSTEROSCOPY,RMV FB
PR HYSTEROSCOPY,RMV MYOMA
PR HYSTEROSCOPY,UTERUS,UNL PROC
PR HYSTEROSCOPY,W/ENDO BX
PR HYSTEROSCOPY,W/ENDOMETRIAL ABLATION
PR INDUCED AB BY INJECT+D&C/EVAC
PR INDUCED AB BY VAG SUPP+D&C/EVAC
PR INDUCED ABORTN BY D&C
PR INDUCED ABORTN BY DIL/EVAC
PR LAP,DX SURGICAL ABD W/BIOPSY
PR MED ABORTION INC ALL EX DRUG
PR NEEDLE BIOPSY, LYMPH NODE(S)
PR PUNCH BIOPSY SKIN SINGLE LESION
PR SIGMOIDOSCOPY,BIOPSY
PR SONO GUIDE NEEDLE BIOPSY
PR SURG RX INCOMPLETE ABORTN
PR SURG RX MISSED ABORTN,1ST TRI
PR SURG RX MISSED ABORTN,2ND TRI
PR TANGENTIAL BIOPSY SKIN EA SEP/ADDITIONAL LESION
PR TANGENTIAL BIOPSY SKIN SINGLE LESION
RETRIEVAL OF EGG OVUM UNDER ANESTHESIA
THERAPEUTIC ABORTION
THERAPEUTIC ABORTION, EVACAUTION OF GESTATION
US BIOPSY PROCEDURE NOT LISTED (RADIOLOGY PERFORMED)
US GUIDED BREAST CORE BIOPSY, LEFT
US GUIDED BREAST CORE BIOPSY, RIGHT
US GUIDED BREAST CORE BIOPSY, RIGHT (S)
US THYROID/PARATHYROID BIOPSY
VAGINAL DILATION & EVACUATION; LABOR AND DELIVERY
VULVA /PERINEUM BIOPSY
VULVAR BIOPSY

IUD insertion common phrases: Common words or phrases in the narrative description in a clinical note about the IUD insertion procedure

betadine
cervical culture
cervix cleaned
cervix cleansed
cervix was cleaned
cervix was cleansed
cleaned cervix
cleansed cervix
consent
intra-uterine device
intrauterine contraception
intrauterine contraceptive
intrauterine device
iud
preg test
pregnancy test

© 2026 Roger JM et al. *JAMA Network Open*.

sounded depth
sounded uterus
speculum
strings trimmed
strings were trimmed
tenaculum
trimmed strings
urine hcg
uterus sounded

Paracervical block medications: Medications that could indicate a paracervical block occurred
LIDOCAINE (PF) 10 MG/ML (1 %) INJECTION SOLUTION
LIDOCAINE (PF) 20 MG/ML (2 %) INJECTION SOLUTION
LIDOCAINE 1 %-EPINEPHRINE 1:100,000 INJECTION SOLUTION
LIDOCAINE 20 MG/ML (2 %)-EPINEPHRINE 1:100,000 INJECTION SOLUTION
LIDOCAINE HCL 10 MG/ML (1 %) INJECTION SOLUTION
LIDOCAINE HCL 20 MG/ML (2 %) INJECTION SOLUTION
LIDOCAINE-EPINEPHRINE (PF) 1 %-1:200,000 INJECTION SOLUTION
LIDOCAINE-EPINEPHRINE 0.5 %-1:200,000 INJECTION SOLUTION

Paracervical block procedures: Procedures that could indicate a paracervical block occurred
PR INJECTION AA&/STRD PARACERVICAL NERVE
PR LIDOCAINE INJECTION

© 2026 Roger JM et al. *JAMA Network Open*.
