# Supplementary material for: Use of Paracervical Blocks for Patients Who Undergo Intrauterine Device Insertion
Source: JAMA Netw Open. 2026 Apr 21;9(4):e268406. doi: 10.1001/jamanetworkopen.2026.8406 (PMC13100842; doi:10.1001/jamanetworkopen.2026.8406)
Supplement: Supplement 2. — Data Sharing Statement [file jamanetwopen-e268406-s002.pdf]

Data Sharing Statement

Roger. Use of Paracervical Blocks for Patients Who Undergo Intrauterine Device Insertion. *JAMA Netw Open*. Published April 21, 2026. doi:10.1001/jamanetworkopen.2026.8406

Data
Data available: No

Additional Information
Explanation for why data not available: The electronic health record data is not publicly available.
